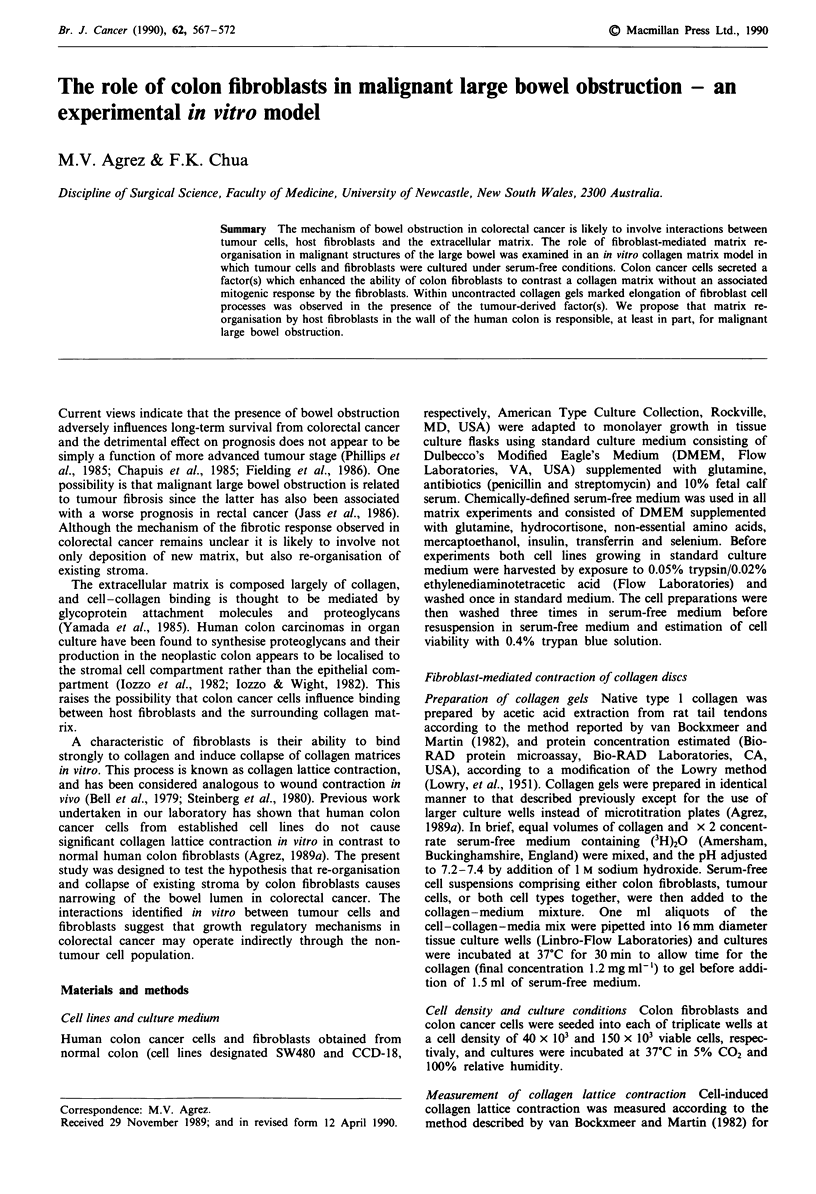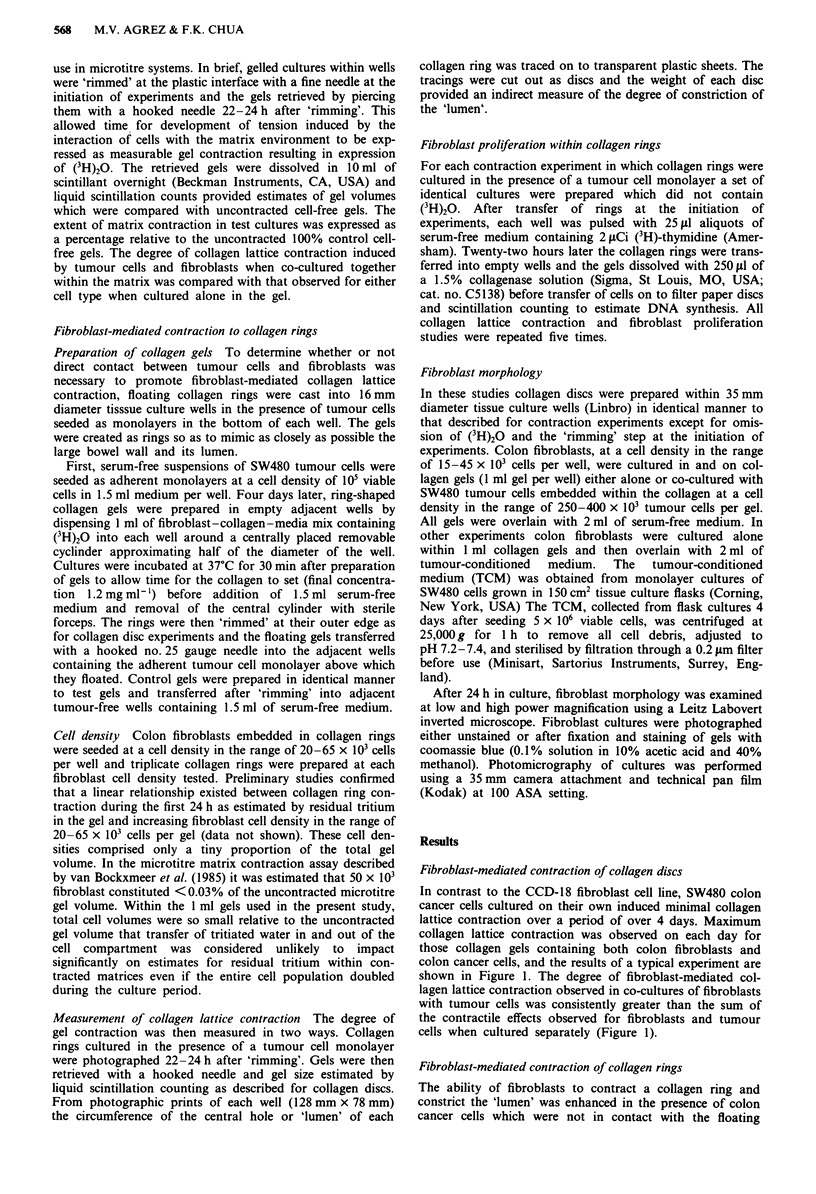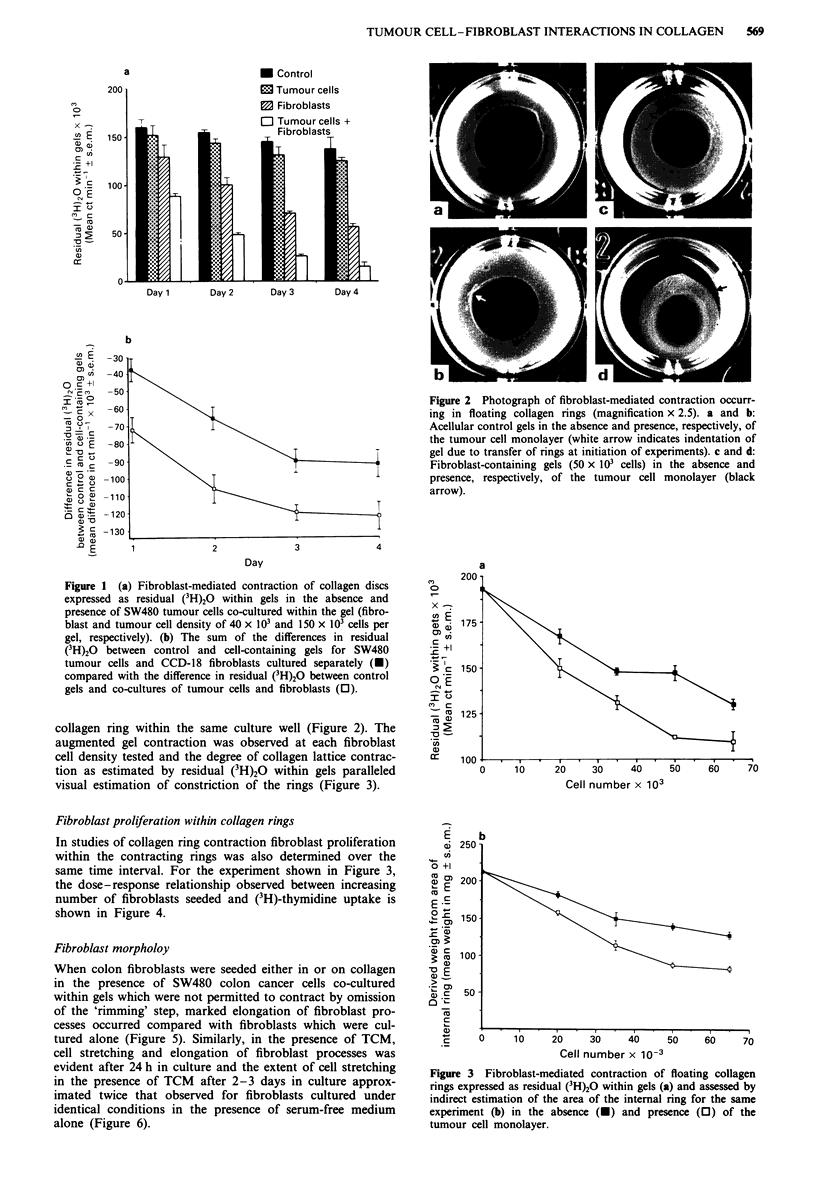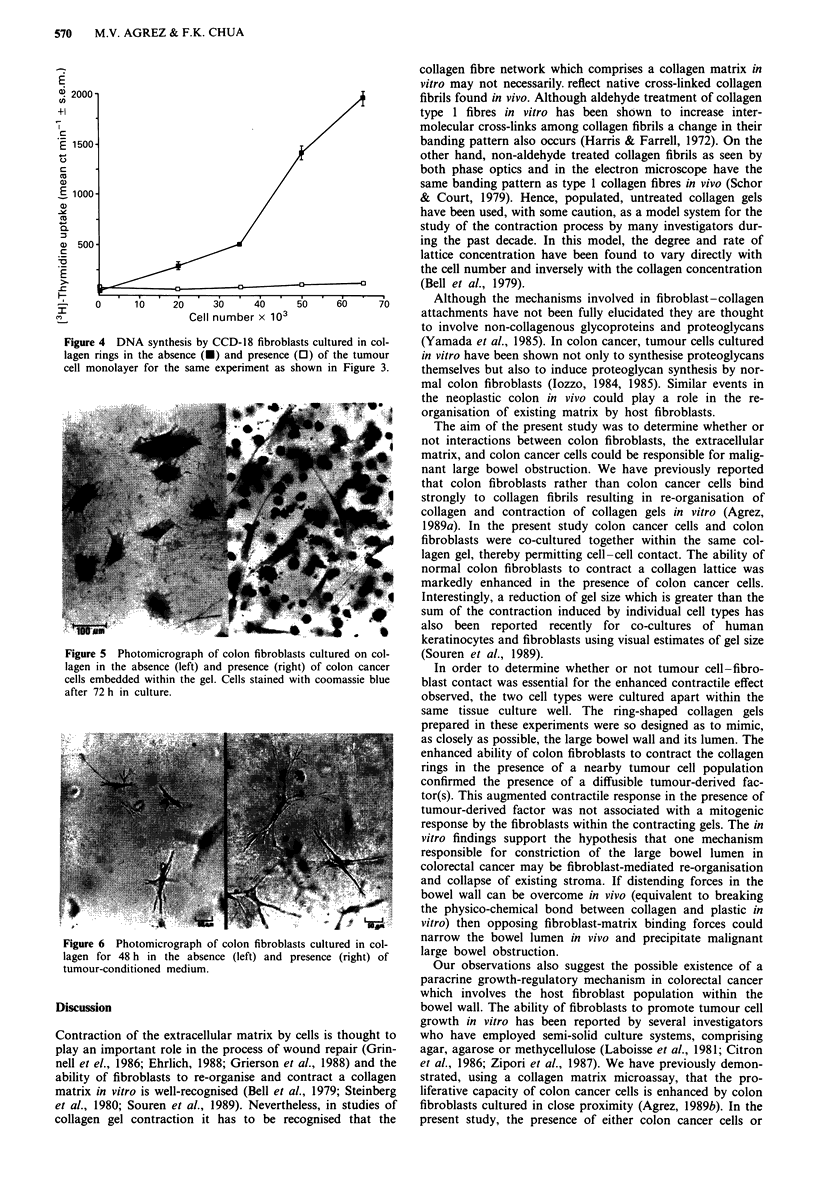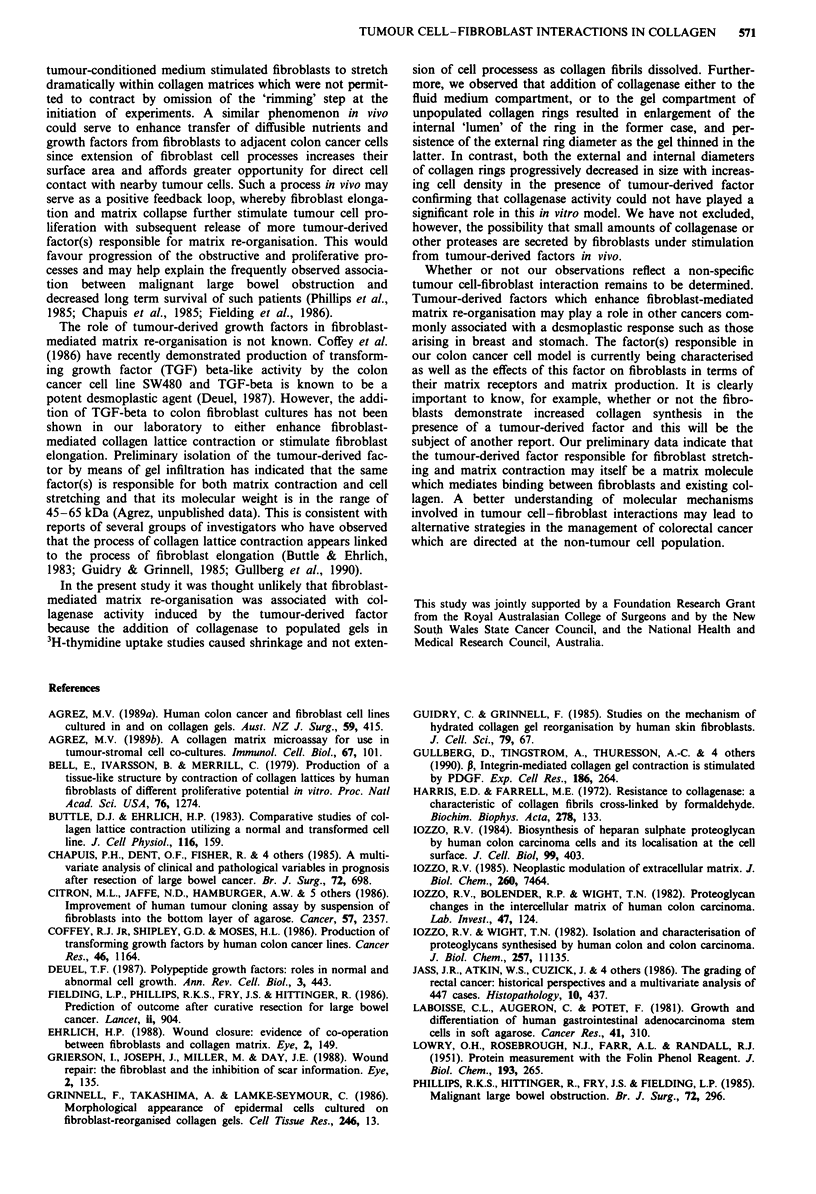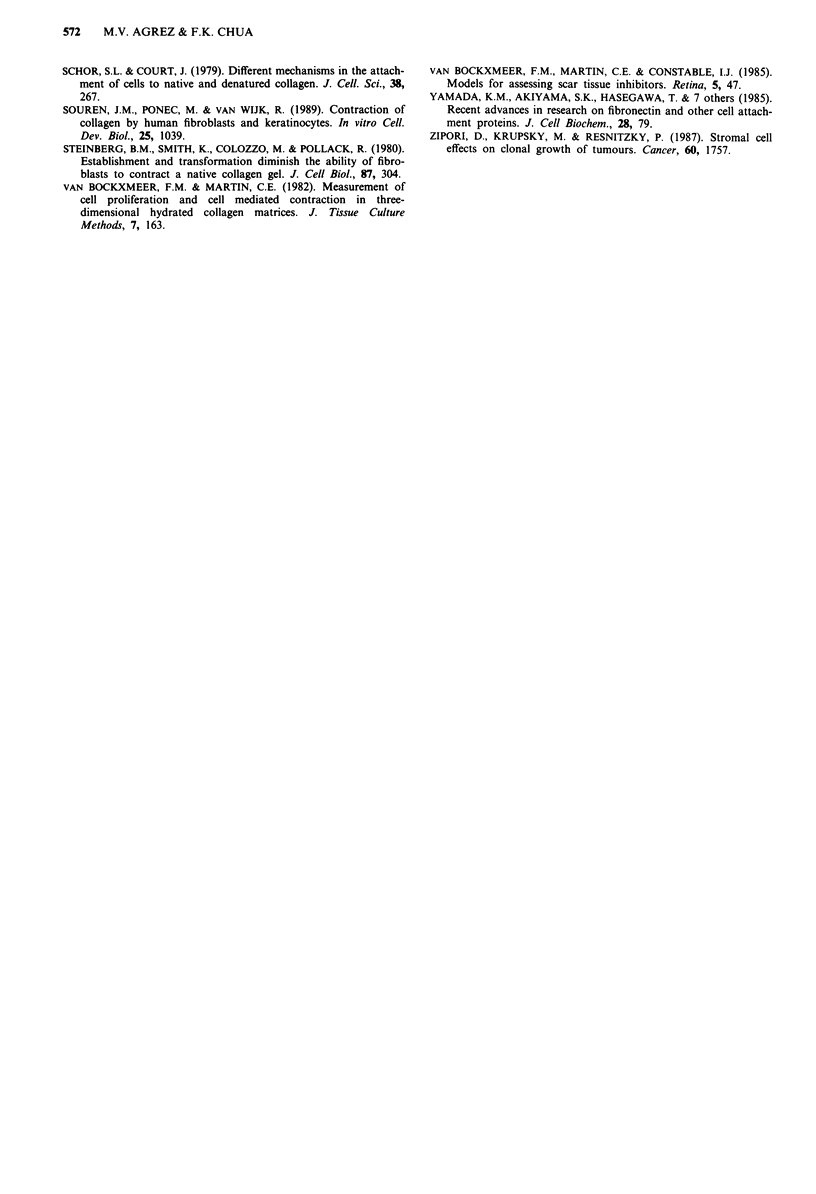# The role of colon fibroblasts in malignant large bowel obstruction--an experimental in vitro model.

**DOI:** 10.1038/bjc.1990.331

**Published:** 1990-10

**Authors:** M. V. Agrez, F. K. Chua

**Affiliations:** Discipline of Surgical Science, Faculty of Medicine, University of Newcastle, New South Wales, Australia.

## Abstract

**Images:**


Br. J. Cancer (1990), 62, 567-572 © Macmillan Press Ltd., 1990

### The role of colon fibroblasts in malignant large bowel obstruction – an experimental *in vitro* model

M.V. Agrez & F.K. Chua

*Discipline of Surgical Science, Faculty of Medicine, University of Newcastle, New South Wales, 2300 Australia.*

**Summary** The mechanism of bowel obstruction in colorectal cancer is likely to involve interactions between tumour cells, host fibroblasts and the extracellular matrix. The role of fibroblast-mediated matrix re-organisation in malignant structures of the large bowel was examined in an *in vitro* collagen matrix model in which tumour cells and fibroblasts were cultured under serum-free conditions. Colon cancer cells secreted a factor(s) which enhanced the ability of colon fibroblasts to contrast a collagen matrix without an associated mitogenic response by the fibroblasts. Within uncontracted collagen gels marked elongation of fibroblast cell processes was observed in the presence of the tumour-derived factor(s). We propose that matrix re-organisation by host fibroblasts in the wall of the human colon is responsible, at least in part, for malignant large bowel obstruction.

Current views indicate that the presence of bowel obstruction adversely influences long-term survival from colorectal cancer and the detrimental effect on prognosis does not appear to be simply a function of more advanced tumour stage (Phillips *et al.*, 1985; Chapuis *et al.*, 1985; Fielding *et al.*, 1986). One possibility is that malignant large bowel obstruction is related to tumour fibrosis since the latter has also been associated with a worse prognosis in rectal cancer (Jass *et al.*, 1986). Although the mechanism of the fibrotic response observed in colorectal cancer remains unclear it is likely to involve not only deposition of new matrix, but also re-organisation of existing stroma.

The extracellular matrix is composed largely of collagen, and cell–collagen binding is thought to be mediated by glycoprotein attachment molecules and proteoglycans (Yamada *et al.*, 1985). Human colon carcinomas in organ culture have been found to synthesise proteoglycans and their production in the neoplastic colon appears to be localised to the stromal cell compartment rather than the epithelial compartment (Iozzo *et al.*, 1982; Iozzo & Wight, 1982). This raises the possibility that colon cancer cells influence binding between host fibroblasts and the surrounding collagen matrix.

A characteristic of fibroblasts is their ability to bind strongly to collagen and induce collapse of collagen matrices *in vitro*. This process is known as collagen lattice contraction, and has been considered analogous to wound contraction *in vivo* (Bell *et al.*, 1979; Steinberg *et al.*, 1980). Previous work undertaken in our laboratory has shown that human colon cancer cells from established cell lines do not cause significant collagen lattice contraction *in vitro* in contrast to normal human colon fibroblasts (Agrez, 1989*a*). The present study was designed to test the hypothesis that re-organisation and collapse of existing stroma by colon fibroblasts causes narrowing of the bowel lumen in colorectal cancer. The interactions identified *in vitro* between tumour cells and fibroblasts suggest that growth regulatory mechanisms in colorectal cancer may operate indirectly through the non-tumour cell population.

### Materials and methods

### *Cell lines and culture medium*

Human colon cancer cells and fibroblasts obtained from normal colon (cell lines designated SW480 and CCD-18, respectively, American Type Culture Collection, Rockville, MD, USA) were adapted to monolayer growth in tissue culture flasks using standard culture medium consisting of Dulbecco's Modified Eagle's Medium (DMEM, Flow Laboratories, VA, USA) supplemented with glutamine, antibiotics (penicillin and streptomycin) and 10% fetal calf serum. Chemically-defined serum-free medium was used in all matrix experiments and consisted of DMEM supplemented with glutamine, hydrocortisone, non-essential amino acids, mercaptoethanol, insulin, transferrin and selenium. Before experiments both cell lines growing in standard culture medium were harvested by exposure to 0.05% trypsin/0.02% ethylenediaminotetracetic acid (Flow Laboratories) and washed once in standard medium. The cell preparations were then washed three times in serum-free medium before resuspension in serum-free medium and estimation of cell viability with 0.4% trypan blue solution.

### *Fibroblast-mediated contraction of collagen discs*

*Preparation of collagen gels* Native type 1 collagen was prepared by acetic acid extraction from rat tail tendons according to the method reported by van Bockxmeer and Martin (1982), and protein concentration estimated (Bio-RAD protein microassay, Bio-RAD Laboratories, CA, USA), according to a modification of the Lowry method (Lowry, *et al.*, 1951). Collagen gels were prepared in identical manner to that described previously except for the use of larger culture wells instead of microtitration plates (Agrez, 1989*a*). In brief, equal volumes of collagen and × 2 concentrate serum-free medium containing $(^3H)_2O$ (Amersham, Buckinghamshire, England) were mixed, and the pH adjusted to 7.2–7.4 by addition of 1 M sodium hydroxide. Serum-free cell suspensions comprising either colon fibroblasts, tumour cells, or both cell types together, were then added to the collagen–medium mixture. One ml aliquots of the cell–collagen–media mix were pipetted into 16 mm diameter tissue culture wells (Linbro-Flow Laboratories) and cultures were incubated at 37°C for 30 min to allow time for the collagen (final concentration $1.2\ mg\ ml^{-1}$) to gel before addition of 1.5 ml of serum-free medium.

*Cell density and culture conditions* Colon fibroblasts and colon cancer cells were seeded into each of triplicate wells at a cell density of $40 \times 10^3$ and $150 \times 10^3$ viable cells, respectivaly, and cultures were incubated at 37°C in 5% $CO_2$ and 100% relative humidity.

*Measurement of collagen lattice contraction* Cell-induced collagen lattice contraction was measured according to the method described by van Bockxmeer and Martin (1982) for

Correspondence: M.V. Agrez.
Received 29 November 1989; and in revised form 12 April 1990.

use in microtitre systems. In brief, gelled cultures within wells were 'rimmed' at the plastic interface with a fine needle at the initiation of experiments and the gels retrieved by piercing them with a hooked needle 22–24 h after 'rimming'. This allowed time for development of tension induced by the interaction of cells with the matrix environment to be expressed as measurable gel contraction resulting in expression of $(^3H)_2O$. The retrieved gels were dissolved in 10 ml of scintillant overnight (Beckman Instruments, CA, USA) and liquid scintillation counts provided estimates of gel volumes which were compared with uncontracted cell-free gels. The extent of matrix contraction in test cultures was expressed as a percentage relative to the uncontracted 100% control cell-free gels. The degree of collagen lattice contraction induced by tumour cells and fibroblasts when co-cultured together within the matrix was compared with that observed for either cell type when cultured alone in the gel.

*Fibroblast-mediated contraction to collagen rings*

*Preparation of collagen gels* To determine whether or not direct contact between tumour cells and fibroblasts was necessary to promote fibroblast-mediated collagen lattice contraction, floating collagen rings were cast into 16 mm diameter tisssue culture wells in the presence of tumour cells seeded as monolayers in the bottom of each well. The gels were created as rings so as to mimic as closely as possible the large bowel wall and its lumen.

First, serum-free suspensions of SW480 tumour cells were seeded as adherent monolayers at a cell density of $10^5$ viable cells in 1.5 ml medium per well. Four days later, ring-shaped collagen gels were prepared in empty adjacent wells by dispensing 1 ml of fibroblast–collagen–media mix containing $(^3H)_2O$ into each well around a centrally placed removable cyclinder approximating half of the diameter of the well. Cultures were incubated at 37°C for 30 min after preparation of gels to allow time for the collagen to set (final concentration 1.2 mg ml$^{-1}$) before addition of 1.5 ml serum-free medium and removal of the central cylinder with sterile forceps. The rings were then 'rimmed' at their outer edge as for collagen disc experiments and the floating gels transferred with a hooked no. 25 gauge needle into the adjacent wells containing the adherent tumour cell monolayer above which they floated. Control gels were prepared in identical manner to test gels and transferred after 'rimming' into adjacent tumour-free wells containing 1.5 ml of serum-free medium.

*Cell density* Colon fibroblasts embedded in collagen rings were seeded at a cell density in the range of $20-65 \times 10^3$ cells per well and triplicate collagen rings were prepared at each fibroblast cell density tested. Preliminary studies confirmed that a linear relationship existed between collagen ring contraction during the first 24 h as estimated by residual tritium in the gel and increasing fibroblast cell density in the range of $20-65 \times 10^3$ cells per gel (data not shown). These cell densities comprised only a tiny proportion of the total gel volume. In the microtitre matrix contraction assay described by van Bockxmeer *et al.* (1985) it was estimated that $50 \times 10^3$ fibroblast constituted $<0.03\%$ of the uncontracted microtitre gel volume. Within the 1 ml gels used in the present study, total cell volumes were so small relative to the uncontracted gel volume that transfer of tritiated water in and out of the cell compartment was considered unlikely to impact significantly on estimates for residual tritium within contracted matrices even if the entire cell population doubled during the culture period.

*Measurement of collagen lattice contraction* The degree of gel contraction was then measured in two ways. Collagen rings cultured in the presence of a tumour cell monolayer were photographed 22–24 h after 'rimming'. Gels were then retrieved with a hooked needle and gel size estimated by liquid scintillation counting as described for collagen discs. From photographic prints of each well (128 mm × 78 mm) the circumference of the central hole or 'lumen' of each collagen ring was traced on to transparent plastic sheets. The tracings were cut out as discs and the weight of each disc provided an indirect measure of the degree of constriction of the 'lumen'.

*Fibroblast proliferation within collagen rings*

For each contraction experiment in which collagen rings were cultured in the presence of a tumour cell monolayer a set of identical cultures were prepared which did not contain $(^3H)_2O$. After transfer of rings at the initiation of experiments, each well was pulsed with 25 µl aliquots of serum-free medium containing 2 µCi $(^3H)$-thymidine (Amersham). Twenty-two hours later the collagen rings were transferred into empty wells and the gels dissolved with 250 µl of a 1.5% collagenase solution (Sigma, St Louis, MO, USA; cat. no. C5138) before transfer of cells on to filter paper discs and scintillation counting to estimate DNA synthesis. All collagen lattice contraction and fibroblast proliferation studies were repeated five times.

*Fibroblast morphology*

In these studies collagen discs were prepared within 35 mm diameter tissue culture wells (Linbro) in identical manner to that described for contraction experiments except for omission of $(^3H)_2O$ and the 'rimming' step at the initiation of experiments. Colon fibroblasts, at a cell density in the range of $15-45 \times 10^3$ cells per well, were cultured in and on collagen gels (1 ml gel per well) either alone or co-cultured with SW480 tumour cells embedded within the collagen at a cell density in the range of $250-400 \times 10^3$ tumour cells per gel. All gels were overlain with 2 ml of serum-free medium. In other experiments colon fibroblasts were cultured alone within 1 ml collagen gels and then overlain with 2 ml of tumour-conditioned medium. The tumour-conditioned medium (TCM) was obtained from monolayer cultures of SW480 cells grown in 150 cm$^2$ tissue culture flasks (Corning, New York, USA) The TCM, collected from flask cultures 4 days after seeding $5 \times 10^6$ viable cells, was centrifuged at 25,000 *g* for 1 h to remove all cell debris, adjusted to pH 7.2–7.4, and sterilised by filtration through a 0.2 µm filter before use (Minisart, Sartorius Instruments, Surrey, England).

After 24 h in culture, fibroblast morphology was examined at low and high power magnification using a Leitz Labovert inverted microscope. Fibroblast cultures were photographed either unstained or after fixation and staining of gels with coomassie blue (0.1% solution in 10% acetic acid and 40% methanol). Photomicrography of cultures was performed using a 35 mm camera attachment and technical pan film (Kodak) at 100 ASA setting.

### Results

*Fibroblast-mediated contraction of collagen discs*

In contrast to the CCD-18 fibroblast cell line, SW480 colon cancer cells cultured on their own induced minimal collagen lattice contraction over a period of over 4 days. Maximum collagen lattice contraction was observed on each day for those collagen gels containing both colon fibroblasts and colon cancer cells, and the results of a typical experiment are shown in Figure 1. The degree of fibroblast-mediated collagen lattice contraction observed in co-cultures of fibroblasts with tumour cells was consistently greater than the sum of the contractile effects observed for fibroblasts and tumour cells when cultured separately (Figure 1).

*Fibroblast-mediated contraction of collagen rings*

The ability of fibroblasts to contract a collagen ring and constrict the 'lumen' was enhanced in the presence of colon cancer cells which were not in contact with the floating

**Figure 1** (**a**) Fibroblast-mediated contraction of collagen discs expressed as residual $(^3H)_2O$ within gels in the absence and presence of SW480 tumour cells co-cultured within the gel (fibroblast and tumour cell density of $40 \times 10^3$ and $150 \times 10^3$ cells per gel, respectively). (**b**) The sum of the differences in residual $(^3H)_2O$ between control and cell-containing gels for SW480 tumour cells and CCD-18 fibroblasts cultured separately (■) compared with the difference in residual $(^3H)_2O$ between control gels and co-cultures of tumour cells and fibroblasts (□).

collagen ring within the same culture well (Figure 2). The augmented gel contraction was observed at each fibroblast cell density tested and the degree of collagen lattice contraction as estimated by residual $(^3H)_2O$ within gels paralleled visual estimation of constriction of the rings (Figure 3).

*Fibroblast proliferation within collagen rings*

In studies of collagen ring contraction fibroblast proliferation within the contracting rings was also determined over the same time interval. For the experiment shown in Figure 3, the dose-response relationship observed between increasing number of fibroblasts seeded and $(^3H)$-thymidine uptake is shown in Figure 4.

*Fibroblast morpholoy*

When colon fibroblasts were seeded either in or on collagen in the presence of SW480 colon cancer cells co-cultured within gels which were not permitted to contract by omission of the 'rimming' step, marked elongation of fibroblast processes occurred compared with fibroblasts which were cultured alone (Figure 5). Similarly, in the presence of TCM, cell stretching and elongation of fibroblast processes was evident after 24 h in culture and the extent of cell stretching in the presence of TCM after 2-3 days in culture approximated twice that observed for fibroblasts cultured under identical conditions in the presence of serum-free medium alone (Figure 6).

**Figure 2** Photograph of fibroblast-mediated contraction occurring in floating collagen rings (magnification × 2.5). **a** and **b**: Acellular control gels in the absence and presence, respectively, of the tumour cell monolayer (white arrow indicates indentation of gel due to transfer of rings at initiation of experiments). **c** and **d**: Fibroblast-containing gels ($50 \times 10^3$ cells) in the absence and presence, respectively, of the tumour cell monolayer (black arrow).

**Figure 3** Fibroblast-mediated contraction of floating collagen rings expressed as residual $(^3H)_2O$ within gels (**a**) and assessed by indirect estimation of the area of the internal ring for the same experiment (**b**) in the absence (■) and presence (□) of the tumour cell monolayer.

**Figure 4** DNA synthesis by CCD-18 fibroblasts cultured in collagen rings in the absence (■) and presence (□) of the tumour cell monolayer for the same experiment as shown in Figure 3.

**Figure 5** Photomicrograph of colon fibroblasts cultured on collagen in the absence (left) and presence (right) of colon cancer cells embedded within the gel. Cells stained with coomassie blue after 72 h in culture.

**Figure 6** Photomicrograph of colon fibroblasts cultured in collagen for 48 h in the absence (left) and presence (right) of tumour-conditioned medium.

### Discussion

Contraction of the extracellular matrix by cells is thought to play an important role in the process of wound repair (Grinnell *et el.*, 1986; Ehrlich, 1988; Grierson *et al.*, 1988) and the ability of fibroblasts to re-organise and contract a collagen matrix *in vitro* is well-recognised (Bell *et al.*, 1979; Steinberg *et al.*, 1980; Souren *et al.*, 1989). Nevertheless, in studies of collagen gel contraction it has to be recognised that the collagen fibre network which comprises a collagen matrix *in vitro* may not necessarily. reflect native cross-linked collagen fibrils found *in vivo*. Although aldehyde treatment of collagen type 1 fibres *in vitro* has been shown to increase intermolecular cross-links among collagen fibrils a change in their banding pattern also occurs (Harris & Farrell, 1972). On the other hand, non-aldehyde treated collagen fibrils as seen by both phase optics and in the electron microscope have the same banding pattern as type 1 collagen fibres *in vivo* (Schor & Court, 1979). Hence, populated, untreated collagen gels have been used, with some caution, as a model system for the study of the contraction process by many investigators during the past decade. In this model, the degree and rate of lattice concentration have been found to vary directly with the cell number and inversely with the collagen concentration (Bell *et al.*, 1979).

Although the mechanisms involved in fibroblast-collagen attachments have not been fully elucidated they are thought to involve non-collagenous glycoproteins and proteoglycans (Yamada *et al.*, 1985). In colon cancer, tumour cells cultured *in vitro* have been shown not only to synthesise proteoglycans themselves but also to induce proteoglycan synthesis by normal colon fibroblasts (Iozzo, 1984, 1985). Similar events in the neoplastic colon *in vivo* could play a role in the reorganisation of existing matrix by host fibroblasts.

The aim of the present study was to determine whether or not interactions between colon fibroblasts, the extracellular matrix, and colon cancer cells could be responsible for malignant large bowel obstruction. We have previously reported that colon fibroblasts rather than colon cancer cells bind strongly to collagen fibrils resulting in re-organisation of collagen and contraction of collagen gels *in vitro* (Agrez, 1989*a*). In the present study colon cancer cells and colon fibroblasts were co-cultured together within the same collagen gel, thereby permitting cell-cell contact. The ability of normal colon fibroblasts to contract a collagen lattice was markedly enhanced in the presence of colon cancer cells. Interestingly, a reduction of gel size which is greater than the sum of the contraction induced by individual cell types has also been reported recently for co-cultures of human keratinocytes and fibroblasts using visual estimates of gel size (Souren *et al.*, 1989).

In order to determine whether or not tumour cell-fibroblast contact was essential for the enhanced contractile effect observed, the two cell types were cultured apart within the same tissue culture well. The ring-shaped collagen gels prepared in these experiments were so designed as to mimic, as closely as possible, the large bowel wall and its lumen. The enhanced ability of colon fibroblasts to contract the collagen rings in the presence of a nearby tumour cell population confirmed the presence of a diffusible tumour-derived factor(s). This augmented contractile response in the presence of tumour-derived factor was not associated with a mitogenic response by the fibroblasts within the contracting gels. The *in vitro* findings support the hypothesis that one mechanism responsible for constriction of the large bowel lumen in colorectal cancer may be fibroblast-mediated re-organisation and collapse of existing stroma. If distending forces in the bowel wall can be overcome *in vivo* (equivalent to breaking the physico-chemical bond between collagen and plastic *in vitro*) then opposing fibroblast-matrix binding forces could narrow the bowel lumen *in vivo* and precipitate malignant large bowel obstruction.

Our observations also suggest the possible existence of a paracrine growth-regulatory mechanism in colorectal cancer which involves the host fibroblast population within the bowel wall. The ability of fibroblasts to promote tumour cell growth *in vitro* has been reported by several investigators who have employed semi-solid culture systems, comprising agar, agarose or methycellulose (Laboisse *et al.*, 1981; Citron *et al.*, 1986; Zipori *et al.*, 1987). We have previously demonstrated, using a collagen matrix microassay, that the proliferative capacity of colon cancer cells is enhanced by colon fibroblasts cultured in close proximity (Agrez, 1989*b*). In the present study, the presence of either colon cancer cells or tumour-conditioned medium stimulated fibroblasts to stretch dramatically within collagen matrices which were not permitted to contract by omission of the 'rimming' step at the initiation of experiments. A similar phenomenon *in vivo* could serve to enhance transfer of diffusible nutrients and growth factors from fibroblasts to adjacent colon cancer cells since extension of fibroblast cell processes increases their surface area and affords greater opportunity for direct cell contact with nearby tumour cells. Such a process *in vivo* may serve as a positive feedback loop, whereby fibroblast elongation and matrix collapse further stimulate tumour cell proliferation with subsequent release of more tumour-derived factor(s) responsible for matrix re-organisation. This would favour progression of the obstructive and proliferative processes and may help explain the frequently observed association between malignant large bowel obstruction and decreased long term survival of such patients (Phillips *et al.*, 1985; Chapuis *et al.*, 1985; Fielding *et al.*, 1986).

The role of tumour-derived growth factors in fibroblast-mediated matrix re-organisation is not known. Coffey *et al.* (1986) have recently demonstrated production of transforming growth factor (TGF) beta-like activity by the colon cancer cell line SW480 and TGF-beta is known to be a potent desmoplastic agent (Deuel, 1987). However, the addition of TGF-beta to colon fibroblast cultures has not been shown in our laboratory to either enhance fibroblast-mediated collagen lattice contraction or stimulate fibroblast elongation. Preliminary isolation of the tumour-derived factor by means of gel infiltration has indicated that the same factor(s) is responsible for both matrix contraction and cell stretching and that its molecular weight is in the range of 45-65 kDa (Agrez, unpublished data). This is consistent with reports of several groups of investigators who have observed that the process of collagen lattice contraction appears linked to the process of fibroblast elongation (Buttle & Ehrlich, 1983; Guidry & Grinnell, 1985; Gullberg *et al.*, 1990).

In the present study it was thought unlikely that fibroblast-mediated matrix re-organisation was associated with collagenase activity induced by the tumour-derived factor because the addition of collagenase to populated gels in $^{3}$H-thymidine uptake studies caused shrinkage and not extension of cell processess as collagen fibrils dissolved. Furthermore, we observed that addition of collagenase either to the fluid medium compartment, or to the gel compartment of unpopulated collagen rings resulted in enlargement of the internal 'lumen' of the ring in the former case, and persistence of the external ring diameter as the gel thinned in the latter. In contrast, both the external and internal diameters of collagen rings progressively decreased in size with increasing cell density in the presence of tumour-derived factor confirming that collagenase activity could not have played a significant role in this *in vitro* model. We have not excluded, however, the possibility that small amounts of collagenase or other proteases are secreted by fibroblasts under stimulation from tumour-derived factors *in vivo*.

Whether or not our observations reflect a non-specific tumour cell-fibroblast interaction remains to be determined. Tumour-derived factors which enhance fibroblast-mediated matrix re-organisation may play a role in other cancers commonly associated with a desmoplastic response such as those arising in breast and stomach. The factor(s) responsible in our colon cancer cell model is currently being characterised as well as the effects of this factor on fibroblasts in terms of their matrix receptors and matrix production. It is clearly important to know, for example, whether or not the fibroblasts demonstrate increased collagen synthesis in the presence of a tumour-derived factor and this will be the subject of another report. Our preliminary data indicate that the tumour-derived factor responsible for fibroblast stretching and matrix contraction may itself be a matrix molecule which mediates binding between fibroblasts and existing collagen. A better understanding of molecular mechanisms involved in tumour cell-fibroblast interactions may lead to alternative strategies in the management of colorectal cancer which are directed at the non-tumour cell population.

This study was jointly supported by a Foundation Research Grant from the Royal Australasian College of Surgeons and by the New South Wales State Cancer Council, and the National Health and Medical Research Council, Australia.

### References

AGREZ, M.V. (1989*a*). Human colon cancer and fibroblast cell lines cultured in and on collagen gels. *Aust. NZ J. Surg.*, **59**, 415.

AGREZ, M.V. (1989*b*). A collagen matrix microassay for use in tumour-stromal cell co-cultures. *Immunol. Cell. Biol.*, **67**, 101.

BELL, E., IVARSSON, B. & MERRILL, C. (1979). Production of a tissue-like structure by contraction of collagen lattices by human fibroblasts of different proliferative potential *in vitro*. *Proc. Natl Acad. Sci. USA*, **76**, 1274.

BUTTLE, D.J. & EHRLICH, H.P. (1983). Comparative studies of collagen lattice contraction utilizing a normal and transformed cell line. *J. Cell Physiol.*, **116**, 159.

CHAPUIS, P.H., DENT, O.F., FISHER, R. & 4 others (1985). A multivariate analysis of clinical and pathological variables in prognosis after resection of large bowel cancer. *Br. J. Surg.*, **72**, 698.

CITRON, M.L., JAFFE, N.D., HAMBURGER, A.W. & 5 others (1986). Improvement of human tumour cloning assay by suspension of fibroblasts into the bottom layer of agarose. *Cancer*, **57**, 2357.

COFFEY, R.J. JR, SHIPLEY, G.D. & MOSES, H.L. (1986). Production of transforming growth factors by human colon cancer lines. *Cancer Res.*, **46**, 1164.

DEUEL, T.F. (1987). Polypeptide growth factors: roles in normal and abnormal cell growth. *Ann. Rev. Cell. Biol.*, **3**, 443.

FIELDING, L.P., PHILLIPS, R.K.S., FRY, J.S. & HITTINGER, R. (1986). Prediction of outcome after curative resection for large bowel cancer. *Lancet*, **ii**, 904.

EHRLICH, H.P. (1988). Wound closure: evidence of co-operation between fibroblasts and collagen matrix. *Eye*, **2**, 149.

GRIERSON, I., JOSEPH, J., MILLER, M. & DAY, J.E. (1988). Wound repair: the fibroblast and the inhibition of scar information. *Eye*, **2**, 135.

GRINNELL, F., TAKASHIMA, A. & LAMKE-SEYMOUR, C. (1986). Morphological appearance of epidermal cells cultured on fibroblast-reorganised collagen gels. *Cell Tissue Res.*, **246**, 13.

GUIDRY, C. & GRINNELL, F. (1985). Studies on the mechanism of hydrated collagen gel reorganisation by human skin fibroblasts. *J. Cell. Sci.*, **79**, 67.

GULLBERG, D., TINGSTROM, A., THURESSON, A.-C. & 4 others (1990). $\beta$, Integrin-mediated collagen gel contraction is stimulated by PDGF. *Exp. Cell Res.*, **186**, 264.

HARRIS, E.D. & FARRELL, M.E. (1972). Resistance to collagenase: a characteristic of collagen fibrils cross-linked by formaldehyde. *Biochim. Biophys. Acta*, **278**, 133.

IOZZO, R.V. (1984). Biosynthesis of heparan sulphate proteoglycan by human colon carcinoma cells and its localisation at the cell surface. *J. Cell. Biol*, **99**, 403.

IOZZO, R.V. (1985). Neoplastic modulation of extracellular matrix. *J. Biol. Chem.*, **260**, 7464.

IOZZO, R.V., BOLENDER, R.P. & WIGHT, T.N. (1982). Proteoglycan changes in the intercellular matrix of human colon carcinoma. *Lab. Invest.*, **47**, 124.

IOZZO, R.V. & WIGHT, T.N. (1982). Isolation and characterisation of proteoglycans synthesised by human colon and colon carcinoma. *J. Biol. Chem.*, **257**, 11135.

JASS, J.R., ATKIN, W.S., CUZICK, J. & 4 others (1986). The grading of rectal cancer: historical perspectives and a multivariate analysis of 447 cases. *Histopathology*, **10**, 437.

LABOISSE, C.L., AUGERON, C. & POTET, F. (1981). Growth and differentiation of human gastrointestinal adenocarcinoma stem cells in soft agarose. *Cancer Res.*, **41**, 310.

LOWRY, O.H., ROSEBROUGH, N.J., FARR, A.L. & RANDALL, R.J. (1951). Protein measurement with the Folin Phenol Reagent. *J. Biol. Chem.*, **193**, 265.

PHILLIPS, R.K.S., HITTINGER, R., FRY, J.S. & FIELDING, L.P. (1985). Malignant large bowel obstruction. *Br. J. Surg.*, **72**, 296.

SCHOR, S.L. & COURT, J. (1979). Different mechanisms in the attachment of cells to native and denatured collagen. *J. Cell. Sci.*, **38**, 267.

SOUREN, J.M., PONEC, M. & VAN WIJK, R. (1989). Contraction of collagen by human fibroblasts and keratinocytes. *In vitro Cell. Dev. Biol.*, **25**, 1039.

STEINBERG, B.M., SMITH, K., COLOZZO, M. & POLLACK, R. (1980). Establishment and transformation diminish the ability of fibroblasts to contract a native collagen gel. *J. Cell Biol.*, **87**, 304.

VAN BOCKXMEER, F.M. & MARTIN, C.E. (1982). Measurement of cell proliferation and cell mediated contraction in three-dimensional hydrated collagen matrices. *J. Tissue Culture Methods*, **7**, 163.

VAN BOCKXMEER, F.M., MARTIN, C.E. & CONSTABLE, I.J. (1985). Models for assessing scar tissue inhibitors. *Retina*, **5**, 47.

YAMADA, K.M., AKIYAMA, S.K., HASEGAWA, T. & 7 others (1985). Recent advances in research on fibronectin and other cell attachment proteins. *J. Cell Biochem.*, **28**, 79.

ZIPORI, D., KRUPSKY, M. & RESNITZKY, P. (1987). Stromal cell effects on clonal growth of tumours. *Cancer*, **60**, 1757.